# Stability of Polyphenols and Antioxidant Activity of Cellulose-Based Encapsulates Enriched with Tart Cherry Juice Polyphenols

**DOI:** 10.3390/molecules31142449

**Published:** 2026-07-13

**Authors:** Josipa Krezić, Ivana Buljeta, Anita Pichler, Mirela Kopjar

**Affiliations:** 1 Faculty of Agriculture and Food Technology, University of Mostar, Biskupa Čule bb, 88000 Mostar, Bosnia and Herzegovina; josipa.krezic@aptf.sum.ba; 2 Faculty of Food Technology Osijek, Josip Juraj Strossmayer University of Osijek, Franje Kuhača 18, 31000 Osijek, Croatia; ivana.buljeta@hotmail.com (I.B.); anita.pichler@ptfos.hr (A.P.)

**Keywords:** tart cherry juice, cellulose, encapsulation, phenolic compounds, anthocyanins, antioxidant activity, storage stability

## Abstract

This study investigated the potential of cellulose as a carrier for bioactive phenolic compounds from tart cherry juice. Using a freeze-drying method, encapsulates were prepared by varying cellulose content (2.5%, 5%, 7.5%, and 10%) and complexation times (15 and 60 min) while maintaining a constant juice volume. The prepared encapsulates were characterized by determining the concentrations of total phenols, monomeric anthocyanins, and proanthocyanidins, alongside antioxidant activity by spectrophotometric methods, individual polyphenols by HPLC, color parameters and structural changes via IR spectroscopy. Additionally, the stability of these parameters was evaluated after one year of storage at room temperature. Results indicated that the highest concentrations of phenolic compounds and the strongest antioxidant potential were achieved with 2.5% cellulose content (the lowest content) after 15 min of complexation (shorter time). Concentrations of polyphenols decreased as the cellulose contents increased. Although changes in chemical parameters occurred during storage, the results confirm that cellulose is a viable carrier for tart cherry phenolics, particularly at lower contents, offering a promising approach for formulating stable bioactive delivery systems.

## 1. Introduction

Phenolic compounds have attracted considerable attention in the scientific community due to their diverse chemical structures and various health-promoting impacts, including antioxidant activity, anti-inflammatory effect, antimicrobial properties, and antiproliferative influence [1]. Fruits represent important natural sources of these bioactive compounds; however, their concentration, chemical form, and stability may limit their bioavailability and consequently their physiological effects. During harvesting, through processing, and storage, phenolic compounds may undergo degradation or interact with other food components, which can further reduce their stability and functionality [2]. Therefore, more research efforts have been directed toward developing strategies to improve the stability and delivery of phenolic compounds in food products.

One promising approach is the preparation of delivery systems based on interactions between phenolic compounds and dietary fibers. Phenolics and dietary fibers are important functional components of plant-based foods and have been associated with various health benefits [3]. Intake of dietary fiber has been correlated with reduced risks of cardiovascular diseases, gastrointestinal disorders, obesity, diabetes, and hypertension [4]. In plant tissues, polyphenols are primarily located in vacuoles but can interact with cell wall polysaccharides, specifically cellulose, hemicellulose, and pectin, and additionally can react with them during processing and digestion [2]. These interactions can influence the stability, availability, and functionality of phenolic compounds in foods. Consequently, the preparation of encapsulates combining dietary fibers and phenolics has been investigated in terms of stability, potential health effects, and their impact on food quality [5]. Cellulose is one of the most abundant natural polysaccharides and a key structural component of plant cell walls [6]. It consists of glucose units linked through β-(1 → 4) and is insoluble in water and most common organic solvents, which contributes to its stability and functionality. Due to its favorable physicochemical properties, cellulose and especially its derivatives are commonly used in the food industry as stabilizers, hydrocolloids, and carrier materials. Cellulose can act as an effective carrier matrix that protects sensitive bioactive compounds and improves their stability. These properties make cellulose a suitable material for encapsulation and delivery applications [7,8]. Therefore, in this study, cellulose was selected as a delivery system for tart cherry phenolics. Additionally, cellulose is an important dietary fiber.

Tart cherries (*Prunus cerasus* L.), belonging to the *Rosaceae* family, are recognized as rich sources of phenolic compounds. According to Mayta-Apaza [9], they are rich in flavonols, anthocyanins, and chlorogenic acid. Due to their high phenolic content and associated antioxidant activity, tart cherries are considered promising raw materials for the development of functional ingredients and delivery systems containing bioactive phenolic compounds.

Most previous research has focused on examining the bioavailability and bioaccessibility of phenols (anthocyanins and phenolic acids) bound to complex plant cell wall components (PCWs) in the gastrointestinal tract under various conditions, as well as the influence of molecular bond types on their interactions [10,11]. Non-covalent interactions, like hydrogen bonding and hydrophobic interactions, are the primary forces causing the interaction between phenolics and PCWs. These interactions are further affected by environmental conditions, such as pH and temperature [10,11,12,13,14,15]. Recent studies on polymer/phenol complexes have explored the adsorption of phenols from various plant sources onto biopolymers and their application in enriching food products [16,17]. However, limited attention has been given to the controlled preparation of cellulose–phenolic complexes and especially their stability during long-term storage, which is essential for their practical application in food products. Through the literature, it can be seen that numerous studies are designed for preparation of delivery systems of phenolic compounds; however, their stability over time is overlooked.

Building on our previous findings regarding cellulose as a carrier for raspberry bioactive compounds [8] and phenolic acids [18], this study aims to investigate the use of cellulose as a carrier for phenolic compounds from tart cherry juice. Hence, encapsulates were prepared by complexation of cellulose and tart cherry juice. Encapsulates were prepared by mixing a fixed juice volume with varying contents of cellulose (2.5–10%) over complexation periods of 15 and 60 min, which was afterwards freeze-dried. In previously mentioned studies [8,18], we observed that the lowest cellulose content was the better choice. In the first of these studies, we investigated the behavior of raspberry juice polyphenols, and we therefore wanted to compare the behavior of polyphenols from a different source, i.e., tart cherry juice, to see whether the same tendency would be observed. The prepared encapsulates were evaluated for total polyphenols, proanthocyanidins and monomeric anthocyanins by spectrophotometric methods, individual polyphenols by HPLC, antioxidant activity, color parameters and structural changes. To determine their shelf-life stability, the samples were maintained at room temperature for 12 months before analysis, and previously mentioned parameters were again determined.

## 2. Results

### 2.1. Polyphenols of Cellulose/Tart Cherry Encapsulates

Table 1 presents the total phenolics, proanthocyanidins and monomeric anthocyanins determined spectrophotometrically in the tart cherry juice used for the preparation of cellulose encapsulates, with contents of 6.58 g/L, 1255.12 mg/L and 469.39 mg/L, respectively. HPLC analysis revealed that the juice contained two anthocyanins, cyanidin-3-glucosyl-rutinoside and cyanidin-3-rutinoside, at concentrations of 344.29 µg/mL and 423.71 µg/mL, respectively; two phenolic acids, neochlorogenic and chlorogenic acids, at concentrations of 105.92 µg/mL and 173.42 µg/mL, respectively; and rutin, quercetin and kaempferol at concentrations of 78.54 µg/mL, 46.25 µg/mL and 31.23 µg/mL, respectively. The pH value of the juice was 3.20, while the total soluble solids content was 8.2%.

**Figure 1:**
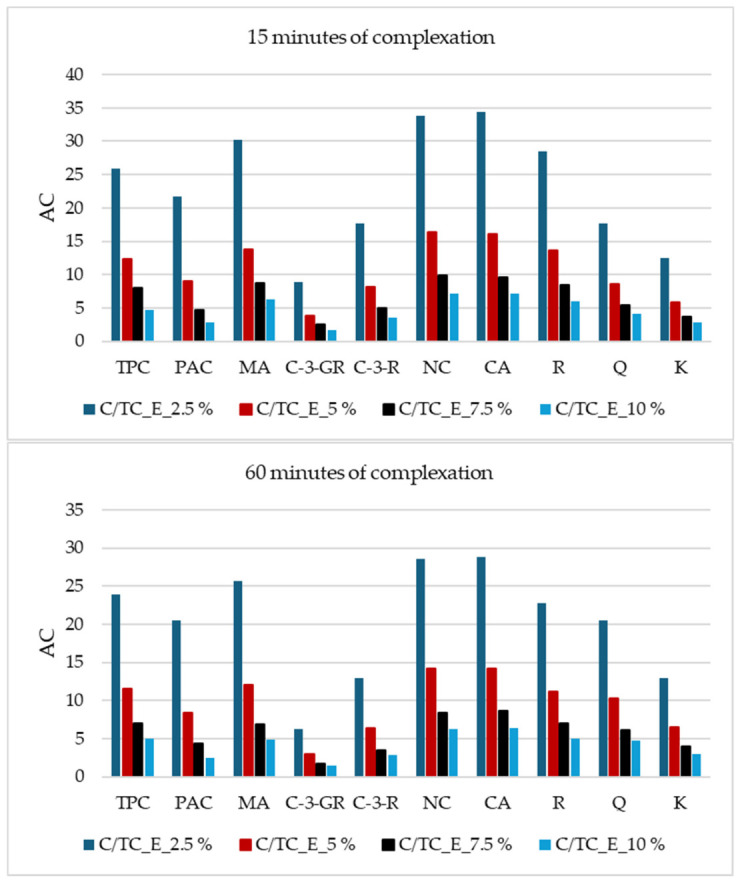
Adsorption capacities of phenolic compounds on cellulose depending on cellulose amount and time of complexation (TPC—total polyphenols, PAC—proanthocyanidins, MA—monomeric anthocyanins, C-3-GR—cyanidin-3-glucosyl-rutinoside, C-3-R—cyanidin-3-rutinoside, NC—neochlorogenic acid, CA—chlorogenic acid, R—rutin, Q—quercetin, K—kaempferol, C/TC_E—encapsulates, 2.5–10—amount of cellulose used for complexation, 15 and 60—min of complexation).

Adsorption capacities (ACs) for phenolic compounds on cellulose (all amounts) and for both complexation times are presented in Figure 1. Results are presented per gram of cellulose. Generally, with increasing cellulose content, a decrease in AC was observed for both complexation times. The ACs of total phenolics ranged from 5% to 26% when complexation was conducted for 15 min and from 5% to 24% when complexation was conducted for 60 min. The same tendency was observed for proanthocyanidins and monomeric anthocyanins. For proanthocyanidins, ACs ranged from 3% to 22% for 15 min of complexation and from 2% to 20% for 60 min of complexation. For monomeric anthocyanins, higher AC values were achieved, from 6% to 30% for 15 min of complexation and from 5% to 26% for 60 min of complexation. Results for individual polyphenols are slightly different. The highest AC values were achieved for chlorogenic and neochlorogenic acids, ranging from 7% to 34% for 15 min of complexation and lower values from 6% to 29% for 60 min of complexation. For rutin, particularly when 15 min of complexation was applied, high AC values were also achieved, from 6% to 28%, while ACs ranged from 5% to 23% for 60 min of complexation. Higher AC values were achieved for cyanidin-3-rutinoside, then for cyanidin-3-glucosyl-rutinoside, from 3% to 18% and from 3% to 13% for 15 and 60 min of complexation, respectively. For cyanidin-3-glucosyl-rutinoside, AC values were 2% to 9% and 1% to 6% for 15 and 60 min of complexation, respectively. Quercetin and kaempferol were the only phenolics for which higher AC values were achieved with 60 min of complexation, and there were smaller differences between samples complexed with different cellulose amounts. The AC for quercetin ranged from 4% to 18% and for kaempferol from 3% to 12% for 15 min of complexation. For 60 min of complexation, the AC for quercetin ranged from 5% to 21% and for kaempferol from 3% to 13%.

Total phenolics, monomeric anthocyanins, and proanthocyanidins contents of tart cherry juice adsorbed on cellulose, both after preparation and following storage, are shown in Table 2, Table 3 and Table 4. In general, the results indicate that both the time of complexation and the content of cellulose significantly influenced the investigated parameters. In the cellulose/tart cherry encapsulates (C/TC_Es) after preparation, the highest total phenolics content (Table 2) had the C/TC_E_2.5% encapsulate formulated for 15 and 60 min (4.26 g/kg and 3.94 g/kg, respectively), followed by encapsulates with higher contents of cellulose (5%, 7.5% and 10%) in which the content of total phenolics gradually decreased. Encapsulate C/TC_E_10% had the lowest phenolics content (3.14 g/kg and 3.26 g/kg) with the times of complexation for 15 and 60 min, respectively. The total content of adsorbed anthocyanins (Table 3) was the highest in the encapsulate C/TC_E_2.5% (354.25 mg/kg) formulated by 15 min of complexation. By increasing the content of cellulose from 2.5% to 10%, the content of anthocyanins adsorbed on cellulose gradually decreased so that encapsulates with 10% cellulose had the lowest contents of anthocyanins (294.25 mg/kg) in samples with a shorter time of complexation (15 min) and 228.21 mg/kg in encapsulates with a longer time of complexation (60 min). This tendency was also recorded for proanthocyanidins content (Table 4). Encapsulates with the lowest cellulose content (2.5%) and a time of complexation of 15 min had the highest content of proanthocyanidins (681.84 mg/kg). By increasing the cellulose content from 2.5% to 10%, the proanthocyanidins contents also decreased so that the lowest contents were determined in the C/TC_E_10% encapsulates (358.45 mg/kg and 306.29 mg/kg, respectively).

To assess the long-term stability and the degree of component retention, the samples were subjected to 12 months of storage at room temperature. The stability of total phenolics, anthocyanins, and proanthocyanidins was evident throughout the storage period, as the initial trends were maintained. Specifically, the encapsulates formulated with the lowest cellulose content (2.5%) and a shorter complexation time (15 min) yielded the highest contents of polyphenols, reaching 3.99 g/kg for total phenolics, 212.49 mg/kg for anthocyanins, and 478.34 mg/kg for proanthocyanidins. The highest retention of total phenolics (about 93%) was determined in the encapsulates containing 2.5% and 10% cellulose with a 15 min complexation period. In contrast, the lowest stability was exhibited by the C/TC_E_5% encapsulate (15 min complexation), which showed a retention of 76% for total phenolics. Retention of anthocyanins was the highest in the encapsulates with 7.5% cellulose (65%), while the lowest retention was in the encapsulates with 10% cellulose (55%) with a longer time of complexation. Anthocyanins retention in cellulose/tart cherry encapsulates with shorter complexation times ranged from 56% (C/TC_E_10%) to 60% (C/TC_E_2.5%). The retention of proanthocyanidins in the encapsulates after storage followed a specific trend. The highest stability was observed in the C/TC_E_2.5%, with retention rates of 70% and 67% for 15 and 60 min of complexation, respectively. In contrast, the lowest retention was recorded in the encapsulates with higher cellulose content, specifically the C/TC_E_7.5% formulated for 60 min (55%) and the C/TC_E_10% formulated for 15 min (56%).

Individual phenolic compounds of encapsulates prepared with a 15 min complexation time are shown in Table 5, whereas those complexed for 60 min (both after preparation and storage) are provided in Table 6. Among the anthocyanins, cyanidin-3-rutinoside and cyanidin-3-glucosyl-rutinoside were detected. The C/TC_E_2.5% encapsulates formulated by 15 min of complexation had the highest concentration of cyanidin-3-rutinoside (185.89 µg/g) as well as in samples that were formulated by 60 min of complexation (135.66 µg/g). The concentration of cyanidin-3-glucosyl-rutinoside was the highest in the C/TC_E_2.5% formulated by 15 min of complexation (76.01 µg/g). Chlorogenic acid was determined in the highest concentration. Encapsulates C/TC_E_2.5% formulated by 15 min of complexation had the highest concentration of chlorogenic acid (151.71 µg/g), while in encapsulates formulated by 60 min of complexation the concentration of chlorogenic acid was 127.21 µg/g. The highest concentrations of other phenolic components (neochlorogenic acid, rutin, quercetin, and kaempferol) were determined also in samples with a shorter time of complexation (15 min) and with the lowest cellulose content (2.5%). The concentration of neochlorogenic acid was 87.03 µg/g, rutin 54.49 µg/g, quercetin 19.92 µg/g, and kaempferol 9.40 µg/g. The concentration of individual phenolics gradually decreased by increasing the cellulose concentration from 2.5% to 10%. An exception is the concentration of cyanidin-3-glucosyl-rutinoside in the samples with the time of complexation of 60 min, where the sample C/TC_E_7.5% had the lowest concentration.

After storage, the concentrations of cyanidin-3-rutinoside and cyanidin-3-glucosyl-rutinoside were the highest in the C/TC_E_2.5% formulated by 15 min of complexation (87.83 µg/g of cyanidin-3-rutinoside and 32.31 µg/g of cyanidin-3-glucosyl-rutinoside). In samples formulated by 60 min of complexation, the concentration of cyanidin-3-rutinoside was 55.02 µg/g, while cyanidin-3-glucosyl-rutinoside was 23.02 µg/g. With increasing cellulose content, from 2.5% to 10%, their concentrations decreased in the samples. Thus, in the C/TC_E_10% sample, the concentrations of cyanidin-3-rutinoside of 53.88 µg/g and cyanidin-3-glucosyl-rutinoside of 21.28 µg/g were determined using 15 min of complexation and cyanidin-3-rutinoside of 46.32 µg/g and cyanidin-3-glucosyl-rutinoside of 20.79 µg/g using 60 min of complexation. The retention of cyanidin-3-rutinoside was the highest in the C/TC_E_2.5% encapsulates using 15 min of complexation (47%), while the lowest retention was in the C/TC_5% encapsulates (37%) using 60 min of complexation. The retention of cyanidin-3-glucosyl-rutinoside ranged from 37% to 49%. In the stored samples, chlorogenic acid was present in the highest concentration, in the range of 136.14 µg/g (C/TC_E_2.5%) to 103.45 µg/g (C/TC_E_10%) in samples formulated by 15 min of complexation, and in the range of 106.93 µg/g (C/TC_E_2.5%) to 96.39 µg/g (C/TC_E_10%) when prolonged complexation was used. Neochlorogenic acid and rutin were also highest in the C/TC_E_2.5%, formulated by 15 min of complexation (76.00 µg/g for neochlorogenic acid and 49.18 µg/g for rutin). Stability of chlorogenic acid in stored samples ranged from 81% to 94%, neochlorogenic acid ranged from 79% to 87%, and rutin ranged from 85% to 94%. The concentration of quercetin in the stored samples was about 22 µg/g, and kaempferol was about 9 µg/g. The percentage of retention was 100%.

### 2.2. Total Antioxidant Activity of Cellulose/Tart Cherry Encapsulates

The antioxidant activity of the cellulose/tart cherry encapsulates was determined by the DPPH, ABTS, FRAP and CUPRAC methods (Table 7). C/TC_Es with a complexation time of 60 min with a cellulose content of 2.5% had the highest antioxidant activity, applying the DPPH method. The values of antioxidant activity ranged from 30.24 to 27.92 µmol TE/100 g. By reducing the content of cellulose from 10% to 2.5%, the values of the antioxidant activity of the encapsulates also decreased. The antioxidant activity of the C/TC_E according to the ABTS, FRAP and CUPRAC methods was the highest in encapsulates with 2.5% cellulose but with a shorter (15 min) complexation time. According to the ABTS method, the antioxidant activity ranged from 18.98 to 13.85 µmol TE/100 g, according to the FRAP method from 3.04 to 2.33 µmol TE/100 g, and according to the CUPRAC method from 167.58 to 125.69 µmol TE/100 g. After storing all cellulose/tart cherry encapsulates, there was a decrease in antioxidant activity. According to the DPPH method, the antioxidant activities ranged around 24 µmol TE/100 g, so that the retention in the stored encapsulates ranged from 81% to 97%. According to the ABTS method, the value ranged from 10.30 µmol TE/100 g to 14.41 µmol TE/100 g (retention percentage from 65% to 83%), according to the FRAP method, from 1.56 µmol TE/100 g to 2.31 µmol TE/100 g (retention percentage from 62% to 84%) and according to the CUPRAC method from 101.16 µmol TE/100 g up to 138.41 µmol TE/100 g (retention percentage from 79% to 89%).

**Figure 2:**
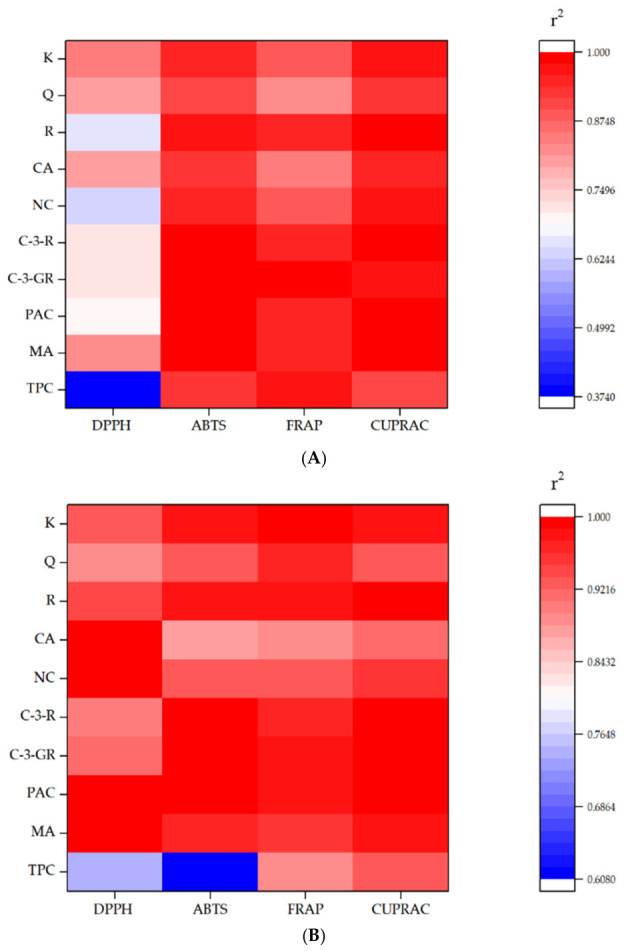
Heat maps of correlations between polyphenols and antioxidant activity of cellulose encapsulates complexed for 15 min after preparation (**A**) and after storage (**B**) (TPC—total polyphenols, PAC—proanthocyanidins, MA—monomeric anthocyanins, C-3-GR—cyanidin-3-glucosyl-rutinoside, C-3-R—cyanidin-3-rutinoside, NC—neochlorogenic acid, CA—chlorogenic acid, R—rutin, Q—quercetin, K—kaempferol).

A positive or negative linear correlation between results can be achieved; the correlation is stronger as the r value approaches +1 or −1, respectively. From the results (Figure 2), for the cellulose encapsulates prepared with 15 min of complexation, only positive correlations were observed when polyphenols were correlated with antioxidant activity. A moderate positive correlation was found between total polyphenol content and antioxidant activity determined by DPPH (0.3753), while strong correlations were observed with other assays (ranging from 0.9087 to 0.9625). The correlation between proanthocyanidin contents and monomeric anthocyanins with antioxidant activities determined by all assays was strongly positive: for proanthocyanidins, r ranged from 0.7066 to 0.9985, and for monomeric anthocyanins, from 0.8153 to 0.9847, with the lowest correlation observed for the DPPH method. Regarding individual anthocyanins, both showed strong positive correlations with antioxidant activity determined by all methods, maintaining the trend that the DPPH method yielded the lowest correlation. Correlation coefficients ranged from 0.7224 to 0.9876 for cyanidin-3-glucosyl-rutinoside and from 0.7236 to 0.9971 for cyanidin-3-rutinoside. For chlorogenic acid, strong correlations with antioxidant activity were observed across all methods (from 0.8036 to 0.9481). For neochlorogenic acid, strong correlations were found with the ABTS, FRAP, and CUPRAC methods (from 0.8941 to 0.9691), and a moderate correlation with DPPH (0.6442). For rutin, the same trend as for neochlorogenic acid was observed: a moderate correlation with the DPPH method (0.6598) and strong correlations with the other methods (from 0.9446 to 0.9923). For quercetin and kaempferol, strong correlations were observed with all methods (quercetin from 0.7923 to 0.9317 and kaempferol from 0.8459 to 0.9595), again with the lowest correlation found using the DPPH method. In Figure 1, the results of the correlation between polyphenols and antioxidant activity for cellulose encapsulates stored for 12 months are also presented. Slightly different results were obtained, with positive correlations retained. In contrast to encapsulates after preparation, a moderate positive correlation was found for the ABTS method (0.6091), while strong correlations were observed with the other methods (ranging from 0.7470 to 0.9244). Strong correlations were found among all methods and for both proanthocyanidins and monomeric anthocyanins (ranging from 0.9838 to 0.9991 and from 0.9577 to 0.9964, respectively). For all individual compounds, strong correlations were obtained with all antioxidant activity methods, ranging from 0.8879 to 0.9997.

**Figure 3:**
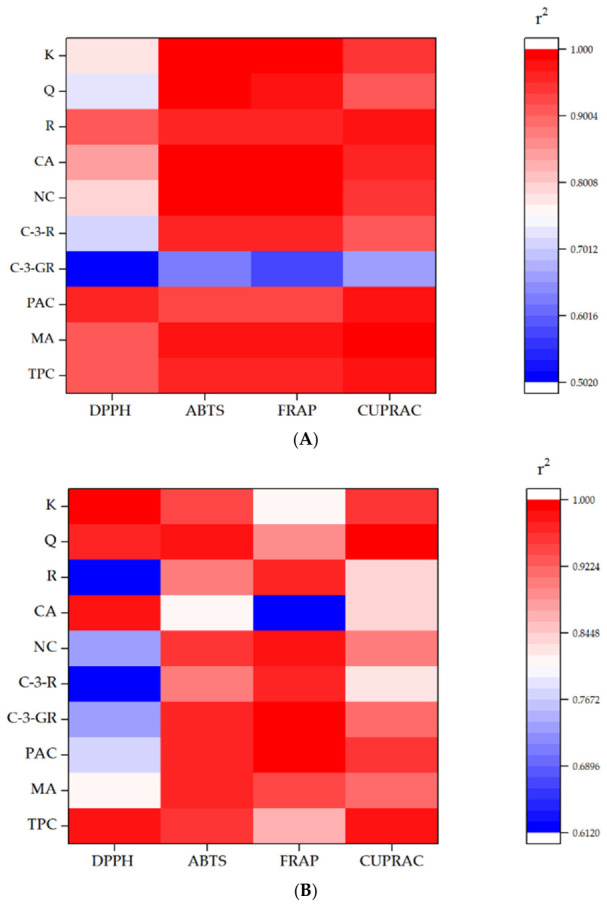
Heat maps of correlations between polyphenols and antioxidant activity of cellulose encapsulates complexed for 60 min after preparation (**A**) and after storage (**B**) (TPC—total polyphenols, PAC—proanthocyanidins, MA—monomeric anthocyanins, C-3-GR—cyanidin-3-glucosyl-rutinoside, C-3-R—cyanidin-3-rutinoside, NC—neochlorogenic acid, CA—chlorogenic acid, R—rutin, Q—quercetin, K—kaempferol).

In Figure 3, the results are presented of cellulose encapsulates prepared with 60 min of complexation after preparation and 12 months of storage. In contrast to cellulose encapsulates complexed for 15 min, those complexed for 60 min showed strong positive correlations among total polyphenols, proanthocyanidins, and monomeric anthocyanins for all methods used to evaluate antioxidant activity (from 0.9071 to 0.9899). Regarding individual compounds, cyanidin-3-glucosyl-rutinoside exhibited only a moderate correlation with all methods (from 0.5033 to 0.6593). All other compounds were strongly positively correlated with all methods (from 0.7069 to 0.9991), but for each compound, the lowest correlation was observed with the DPPH method. The calculation of correlation after storage also revealed that strong correlations were retained among total polyphenols, proanthocyanidins, and monomeric anthocyanins, with all values from all methods (ranging from 0.7002 to 0.9987). Moderate correlations were observed for cyanidin-3-glucosyl-rutinoside and the DPPH method (0.6127), rutin and the DPPH method (0.6247), and chlorogenic acid and the FRAP method (0.6191). For all other compounds with all methods, except those previously mentioned, strong positive correlations were observed (from 0.7325 to 0.9934).

**Figure 4:**
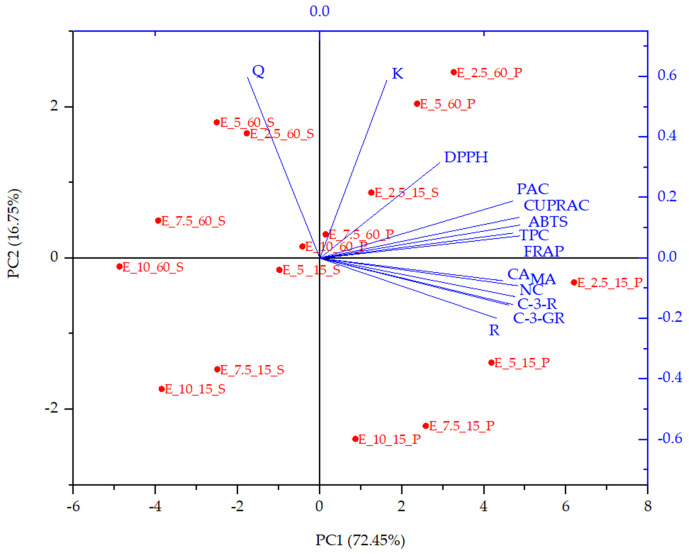
PCA bioplot of all encapsulates based on polyphenols and antioxidant activity (E—encapsulates, 2.5, 5, 7.5 and 10—cellulose content (%), 15 and 60—time of complexation, P—after preparation, S—after storage, TPC—total polyphenols, PAC—proanthocyanidins, MA—monomeric anthocyanins, C-3-GR—cyanidin-3-glucosyl-rutinoside, C-3-R—cyanidin-3-rutinoside, NC—neochlorogenic acid, CA—chlorogenic acid, R—rutin, Q—quercetin, K—kaempferol).

Combining all results from the polyphenols and antioxidant activity evaluations for all encapsulates prepared by complexation for 15 and 60 min, both after preparation and after storage, a PCA plot was created and presented in Figure 4. The PCA plot explains approximately 89% of the total variance, with PC1 accounting for 72.45% and PC2 for 16.75%. Encapsulates were distributed across the biplot, clustering in the directions of similar samples. In the lower right quadrant, defined by positive PC1 and negative PC2, all encapsulates prepared by 15 min of complexation after preparation are located in a diagonal order, corresponding to decreasing concentrations of cyanidin-3-rutinoside, cyanidin-3-glucosyl-rutinoside, neochlorogenic acid, chlorogenic acid, rutin, and monomeric anthocyanins. Encapsulates prepared by 60 min of complexation are located in the upper right quadrant (positive PC1 and positive PC2), except for the sample with 10% cellulose, which is in the upper left quadrant. These samples also followed a diagonal order corresponding to decreasing concentrations of polyphenols. This diagonal order of encapsulates was retained after storage, as a similar trend was observed, with some exceptions, in the concentrations of certain polyphenols. Samples prepared by 15 min of complexation after storage are located in the lower left quadrant (negative PC1 and PC2), with the encapsulate prepared with 2.5% cellulose in the upper right quadrant, making it similar to the samples prepared by 60 min of complexation after preparation. Samples prepared by 60 min of complexation after storage are positioned in the upper left quadrant, with the sample containing 10% cellulose in the lower left quadrant.

### 2.3. Color Parameters of Encapsulates

Table 8 shows the color parameters (L*, a*, b*, °h and C*) for the cellulose/tart cherry encapsulates after preparation, complexed for 15 and 60 min, respectively. The L* value of cellulose was determined to be 93.40, the adsorption of the phenolic components of tart cherry juice led to a decrease in the L* value, making the encapsulates darker. The L* values of the cellulose/tart cherry encapsulate ranged from 66.98 to 71.85. By increasing the content of cellulose from 2.5% to 10%, the L* values increased, that is, the brightness of the encapsulates increased. Cellulose had a positive value of the a* parameter that defines the red tone of the sample (0.42). With the adsorption of tart cherry juice anthocyanins, the a* value increased significantly. In the cellulose/tart cherry encapsulates after preparation and complexation at 15 and 60 min, respectively, the values of the parameter a* were between 25.30 and 29.10. Encapsulates with the highest content of cellulose (10%) had the lowest values of the parameter a*. The b* value defines the yellow tone of the sample, and for cellulose this value was 6.65.

Adsorption of tart cherry juice phenols significantly reduced this value. The b* values for C/TC_Es were between 1.29 and 3.24. Encapsulates with the lowest cellulose content had the highest b* value. By increasing the content of cellulose, the values of the parameter b* decreased. Based on the measured L*, a*, b* values, the color change of the encapsulates after preparation compared to cellulose (ΔE) was calculated. The biggest change in the color of the sample compared to cellulose was found in the complexes with the lowest cellulose content (2.5%) and the shorter complexation time (15 min). ΔE for the C/TC_E_2.5% was determined to be 39.23. By further increasing the content of cellulose, the color change decreased.

The color tone, °h value, for cellulose was 86.34, and the adsorption of tart cherry juice phenols resulted in a significant decrease in the °h value. The °h value for C/TC_E was in the interval from 2.94 to 6.75. The color saturation or C* value for cellulose was 6.66. Adsorption of tart cherry juice phenols led to saturation, mostly for the C/TC_E_2.5% (29.16 and 27.57) encapsulate formulated for 15 min and 60 min, respectively.

Table 9 shows the color parameters of stored cellulose/tart cherry encapsulates. As for the stored samples, the L* values follow the same trend as the L* values for the encapsulates after preparation, a* and b* values were also the highest in the samples with the lowest cellulose content (2.5%), and with an increase in the cellulose content from 2.5% to 10%, their values decreased. The color tone or °h value of stored cellulose/tart cherry encapsulates ranged from 25.37 to 32.91, with the highest value being for the C/TC_E_5% encapsulate. C/TC_E_2.5% (21.50 and 20.36) formulated for 15 min and 60 min respectively had the highest color saturation or C* value. Increasing the content of cellulose resulted in a decrease in color saturation. The color change (ΔE) of the stored cellulose/tart cherry encapsulates compared to the encapsulates after preparation was also determined. The color change in the stored cellulose/tart cherry encapsulates was in the range from 12.61 to 13.55 with complexation times of 15 and 60 min. Generally, it is evident that a decrease in anthocyanin content was accompanied by a decrease in the a* value.

### 2.4. IR Spectra of Encapsulates

**Figure 5:**
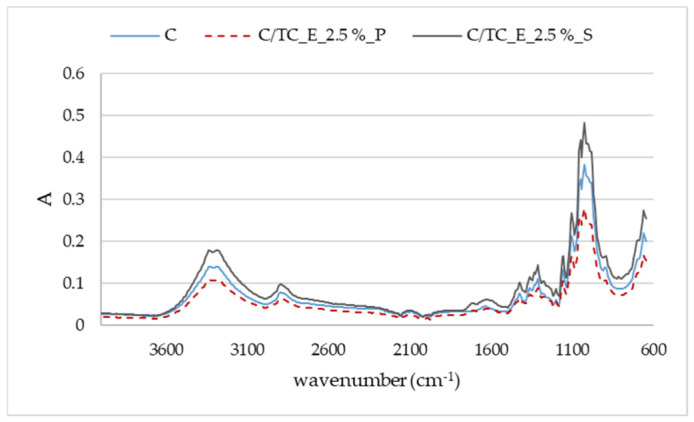
Recorded IR spectra of cellulose (C) and cellulose/tart cherry encapsulates (C/TC__2.5%) after preparation (P) and after storage (S).

The IR spectra of cellulose and cellulose/tart cherry encapsulates, both immediately after preparation and following the storage period, are presented in Figure 5. A significant overlap between the spectra of pure cellulose and the encapsulates was observed at identical wavenumbers. However, in comparison to pure cellulose, the complexes exhibited a distinct new band at 1580 cm^−1^, corresponding to the vibrational stretching of the C–C bond within the phenyl ring. Additionally, two bands appeared in the region between 820 cm^−1^ and 780 cm^−1^, attributed to the C–H ring deformation, which were absent in the spectrum of pure cellulose. The same changes in IR spectra remained after storage of the encapsulates. Structural changes in cellulose caused by the adsorption of polyphenols from tart cherry juices can be confirmed by FTIR-ATR analysis.

## 3. Discussion

The interactions between various dietary fibers, including cellulose, and polyphenolic compounds is governed by their chemical attributes, like molecular weight and structure as well as their physical characteristics and concentrations prior to complexation [19,20]. These components primarily interact through non-covalent mechanisms, involving hydrogen bonding, hydrophobic forces, and van der Waals interactions, or via the physical entrapment of polyphenols within the fiber matrix. Such interactions are significantly modulated by environmental factors, most notably pH levels, temperature, and the duration of the complexation and encapsulation processes [10,11,12,13,14,15]. At the molecular level, the binding of phenolics to cellulose is fundamentally determined by the size and configuration of the phenol molecule [12]. A defining feature of phenolics is the existence of at least one benzene ring and hydroxyl (OH) groups, which facilitate attachment to specific locations on polysaccharide chains. These components mainly bind through hydrogen bonds that are formed between OH groups of the phenolics and the oxygen atoms of the polysaccharides, but also through hydrophobic interactions. Additionally, covalent ester bonds may develop between phenolic acids and polysaccharides [21]. Furthermore, the affinity is influenced by the molecule’s surface properties, size, and porosity. While the pore size of polysaccharides can restrict the diffusion of high-molecular-weight polyphenols [21], some studies have confirmed that larger polyphenolic molecules often bind to cellulose in greater quantities compared to those with lower molecular weights [2].

The complexation mixture contained cellulose and tart cherry juice, so the impact of water during complexation, specifically the adsorption of polyphenols on cellulose, must be considered. Generally, reactions that depend on the diffusion of reactants increase with greater water availability in the mixture. Higher amounts of water strongly affect sensitive compounds such as phenolics, causing their deterioration due to increased molecular mobility and the presence of oxygen, which further enhances oxidation reactions. Among polyphenols, anthocyanins are the most sensitive to water. First, hydrolysis of the glycosidic bond in anthocyanins occurs, resulting in the formation of anthocyanidins, which are more unstable than anthocyanins. The next step in anthocyanin degradation is the opening of the pyrilium ring, leading to the formation of chalcones and brown end products [22]. In addition to the negative impact of water on anthocyanins, these compounds are also known for their stacking effect, that is, additional binding between free anthocyanins and those already attached to cellulose. This effect can contribute to the adsorption of anthocyanins to cellulose, as well as other phenolics, as stated in other studies [10]. However, compounds bonded through the stacking effect were probably the first to degrade during storage, which could explain the lower retention of polyphenols when a lower content of cellulose was used for complexation.

Our findings align with previous research regarding the impact of complexation time on the association of phenolic compounds and cell wall polysaccharides. We observed that extending the complexation time under continuous mixing did not result in enhanced phenolic adsorption onto the cellulose. These interactions typically occur spontaneously within the first minute, followed by a rapid increase within 30 min, after which the binding rate plateaus [2,8,10]. This behavior may be attributed to specific sites on the cellulose molecule that form rigid regions, or to a specific binding affinity threshold; once this level is exceeded, phenolic compounds may form an ionic barrier that prevents further attachment [10]. Costa et al. [23] reported similar results when studying catechin, ferulic acid, and caffeic acid adsorption on xylan and cellulose, noting that while adsorption began in the first minute, active sites on the adsorbent became saturated after only 10 min. According to Liu et al. [3], initial binding is driven by the adsorption of phenols to surface sites on the cellulose molecule, facilitated by labile hydroxyl groups, followed by additional non-covalent stabilization via hydrogen and hydrophobic bonds. Key factors in this non-covalent process include the number of phenolic rings and their conformational flexibility [24].

Given that the adsorption of phenols onto cellulose relies on non-covalent bonding, the prolonged encapsulation time and mechanical mixing likely disrupted these relatively weak hydrogen and hydrophobic links. Consequently, this explains the lower phenolic content found in cellulose/tart cherry encapsulates (C/TC_Es) formulated for 60 min compared to shorter intervals [2,3]. The maximum binding capacity for some compounds, which ranges from 0.4 to 1.4 g per gram of cellulose depending on molecular structure [2], also appears to be a limiting factor. Furthermore, the availability of binding sites on the fibers is crucial. Research on blackberry juice adsorbed onto apple fibers indicated that increasing fiber content above 2% negatively impacted phenolic binding, suggesting that maximum capacity was reached below that content [25]. These trends are consistent with our previous study on raspberry/cellulose encapsulates [8], where samples with lower cellulose content (2.5%) and shorter complexation times (15 min) exhibited higher contents of total phenolics and anthocyanins. Increasing the cellulose content or the mixing duration appears to weaken these rapid interactions, leading to reduced phenolic retention. Additionally, hydrogen bonds could also occur between cellulose molecules resulting in cellulose–cellulose interactions [26,27]. These interactions decrease the number of binding sites for polyphenols and cellulose. Overall, several factors were responsible for the initial adsorption of polyphenols onto cellulose, but all of them were related to the intensity of binding and the molecular structures of the adsorbed compounds and the carrier.

Finally, the interaction between proanthocyanidins and cell wall fibers is influenced by their content, molecular weight, degree of polymerization, and the specific architecture of the cell wall [28]. Higher environmental concentrations of polyphenols have been shown to enhance proanthocyanidin adsorption [14,28]. Structurally, proanthocyanidins containing (+)-catechin units exhibit a stronger affinity for polysaccharides than those with (−)-epicatechin units [28]. While increased cell wall porosity promotes the adsorption of high-molecular-weight tannins, a reduction in porosity—such as that caused by drying—decreases this affinity [19,20,29,30]. In this study, the adsorption of tart cherry proanthocyanidins onto cellulose followed the established trends observed for citrus and apple fibers [25,31,32], with the highest proanthocyanidin contents recorded in encapsulates with the lowest fiber content.

From our results, encapsulates prepared with 2.5% cellulose had the highest antioxidant activity. By increasing the content of cellulose, the antioxidant activity decreased. In the cellulose/tart cherry encapsulates, the values in the stored samples were lower than those after preparation. Also, the antioxidant activity determined by all four methods had different values since the mechanism of action of each method is different. Comparing the selectivity of DPPH radicals and ABTS radical cations, it was observed that DPPH radicals have a higher selectivity in the reaction with a hydrogen donor than ABTS radical cations [33]. A major discrepancy was also found between the metal-reducing assays, as CUPRAC values were significantly higher than FRAP values. The FRAP assay requires a highly acidic medium (pH 3.6), which can alter the phenolics conformation or deactivate the remaining –OH groups, sharply reducing iron (Fe^3+^) reduction. In contrast, CUPRAC operates at a neutral, physiological pH (7.0) [34]. Under these stable conditions, the reagent can better access the complexed tart cherry phenolics, explaining its superior sensitivity and higher values in this system. Additionally, Apak et al. [35] demonstrated that in addition to molecular conjugation, the number and position of free hydroxyl (–OH) groups are directly related to antioxidant activity in the CUPRAC method.

The antioxidant capacity of fiber/phenol complexes has been extensively documented in previous literature [18,25,36,37]. For instance, cellulose/gallic acid and cellulose/caffeic acid systems exhibited peak antioxidant activities at the lowest cellulose content (2.5%). Similarly, blackberry juice polyphenols encapsulated within citrus and apple fiber matrices showed superior antioxidant potential when formulated with 1% fiber content, significantly outperforming complexes with 4% and 10% fiber content, respectively [25,36]. Additionally, Da Rosa et al. [37] reported varying efficiencies in blackberry polyphenol microcapsules, where xanthan (90.75%) and β-cyclodextrin (84.43%) coatings yielded higher antioxidant activities compared to chitosan (80.38%). Their findings indicated that antioxidant performance was directly proportional to the polyphenolic load within the microcapsules.

Recent studies [33,36] demonstrated a decrease in IR spectrum intensity following the adsorption of blackberry polyphenols onto apple and citrus fibers. Our previous study [8] on the adsorption of raspberry polyphenols onto cellulose, as well as the present study on the adsorption of tart cherry polyphenols, showed a decrease in IR spectrum intensity compared to pure cellulose. Buljeta et al. [32] also confirmed, through FTIR analysis, that structural changes occur during the adsorption of quercetin onto apple and citrus fibers, with hydrogen bonding and hydrophobic interactions assumed to be the driving forces behind the interaction. Moon et al. [38] encapsulated quercetin onto soy/chitosan polysaccharides and observed changes in the IR spectrum, suggesting that hydrophobic interactions were the main driving force of quercetin encapsulation. In the study of Savić et al. [39], where quercetin was encapsulated in (2-hydroxypropyl)-β-cyclodextrin, the carrier bands were more dominant in the spectrum than the bands of the carrier and quercetin, which was also the case in our research. Kopjar et al. [18] demonstrated that adsorption of phenolic acids onto cellulose leads to changes in IR spectra. Complexation of cellulose with gallic or caffeic acid resulted in an increase in IR spectrum intensity compared to pure cellulose. Since gallic and caffeic acids contain phenolic –OH groups and one carboxyl group, their structure allows for hydrogen bonding interactions. FTIR analysis confirmed that hydrogen bonding was the primary mechanism of interaction between phenolic acids and cellulose. Other studies have also confirmed that changes in IR spectra are evident after the binding of phenolic compounds to cellulose [8]. In the study by Abdelwahab and Amin [40], a decrease in band intensity on IR spectra was observed after the adsorption of phenols from aqueous solutions onto *Luffa cylindrica* fibers. The authors emphasized that the functional groups on the fiber surface became occupied by phenolic molecules and that phenols penetrated into the interlayer spaces of the fibers.

Cellulose, as a carrier of polyphenols, in this study for tart cherry juice polyphenols, can enable the preparation of functional food additives and expand the field of application of this type of product through the development of new products as well as the improvement of existing ones. Cellulose encapsulates could be used particularly in bakery products, which vary by type, but also in dairy products and fruit and vegetable products. This method of preparing functional food additives represents a green approach, combining plant-based materials into one, so foods to which they are added could be enriched with both cellulose and polyphenols, both known for their health benefits. These additives could also improve the shelf-life stability of products to which they are added due to their antioxidant content, and could modify color. Another aspect that should also be taken into account is if cellulose is extracted from fruit-waste material such as fruit pomace, this type of additive can be considered an upcycled product, contributing to the circular economy.

## 4. Materials and Methods

### 4.1. Materials

Tart cherries (*Prunus cerasus* var. *Oblačinska*) were harvested at the location 46°18′11.0″ N 16°33′14.3″ E near Varaždin (Croatia) in their ripe stage. Cellulose (microcrystalline) was purchased from the manufacturer Kemika (Zagreb, Croatia). Potassium chloride, sodium acetate, ethanol, and ammonium acetate were purchased from Gram-mol d.d. (Zagreb, Croatia). Potassium sodium tartrate tetrahydrate, sodium carbonate, sodium hydroxide, and acetic acid were purchased from T.T.T. (Holy Sunday, Croatia). Trolox, 4-(dimethylamino)-cinnamaldehyde, 2,2-diphenyl-1-picrylhydrazyl (DPPH), 2,2′-azinobis(3-ethylbenzthiazoline-sulfonic acid) (ABTS), ferric chloride and gallic acid were from Sigma-Aldrich (St. Louis, MO, USA). Folin–Ciocalteu reagent was purchased from Carlo Erba Reagents (Sabadell, Spain), while hydrochloric acid was purchased from Panreac (Barcelona, Spain). Standards of ellagic acid, rutin, quercetin, kaempferol, cyanidin-3-glucoside, cyanidin-3-sophoroside, cyanidin-3-rutinoside, neochlorogenic acid, cyanidin-3-O-glucoside chloride, as well as procyanidin B2, were purchased from Extrasynthesis (Genay, France). Chlorogenic acid, copper (II) chloride, neocuproic hemihydrate, and 2,4,6-tripyridyl-s-tyrazine (TPTZ) were purchased from Acros Organics (Geel, Belgium). Ortho-phosphoric acid was purchased from Fisher Scientific (Loughborough, UK) and methanol from J.T. Baker (Deventer, The Netherlands).

### 4.2. Preparation of Cellulose/Tart Cherry Encapsulates

Firstly, tart cherry fruits were washed and their pits were removed. Afterwards, fruits were pressed and the obtained mass was filtered through cheesecloth to obtain tart cherry juice. The juices prepared in this way were thermally treated at 90 °C for 3 min to inactivate the enzymes. To prepare the cellulose/tart cherry encapsulates, cellulose (2.5%, 5%, 7.5%, and 10%) and tart cherry juice were mixed for 15 min or 60 min on a magnetic stirrer at room temperature in appropriate proportions. In 50 mL of juice, 1.25 g, 2.5 g, 3.75 g and 5 g of cellulose were added to perform complexation, and this procedure was done three times. After that, the obtained mixture was centrifuged for 15 min at 4000 rpm. The solid part was separated from the liquid part by centrifugation, and the wet solid part was separated to prepare dry powder, i.e., dry cellulose/tart cherry encapsulates. Cellulose/tart cherry encapsulates were obtained by lyophilization. Before the lyophilization procedure, the wet solid part obtained by centrifugation was frozen at −18 °C for 24 h, and the lyophilization was carried out in a lyophilizer (Christ Freeze Dryer, Alpha 1-4, Osterode am Harz, Germany). The lyophilization conditions were adjusted so that the freezing temperature was −55 °C; the sublimation temperature was from −35 °C to 0 °C under a vacuum of 0.220 mbar; and in the final stage, the isothermal desorption temperature was from 0 °C to 22 °C under a vacuum of 0.060 mbar. The obtained lyophilized samples were immediately used to determine the defined parameters.

### 4.3. Evaluation of Storage Stability

Evaluation of long-term stability, specifically 12-month stability, was conducted. Prepared dried encapsulates were placed in multi-layer plastic laminate foil (polyamide/polyethylene) and vacuum sealed (vacuum sealer by Gorenje, Velenje, Slovenia). The obtained packages were stored for one year at room temperature (25 ± 2 °C) under light to simulate conditions on store shelves.

### 4.4. Extraction of Encapsulates

To prepare the extract, 0.8 g of a dry sample of the cellulose/tart cherry encapsulate was weighed, and 5 mL of methanol acidified with hydrochloric acid (HCl:methanol = 1:99) was added. Extraction was carried out in an ultrasonic bath for 15 min, after which the mixture was left to rest for an additional 15 min to separate the solid and liquid phases. The liquid phase was decanted and then centrifuged for 10 min at 10,000 rpm. The obtained clear extract was put into a plastic test tube. Extraction of the remaining solid phase with a fresh amount of solvent (5 mL) and separation of the phases was repeated three more times in the previously described manner. The obtained extracts were used to determine total phenols, anthocyanins, proanthocyanidins, and antioxidant activity, as well as individual polyphenols, by HPLC.

### 4.5. Determination of Total Phenolic Content

The contents of total phenols were determined using the Folin–Ciocalteu method [41]. A calibration curve was made with gallic acid to express total phenols contents, and the results were expressed as grams of gallic acid per kilogram of sample (g GAE/kg). Briefly, 0.2 mL of sample, 1.8 mL of distilled water, 10 mL of Folin–Ciocalteu reagent (1:10), and 8 mL of 7.5% sodium carbonate solution (Na_2_CO_3_) were pipetted into a test tube, shaken, and left to stand for 2 h in a dark place at room temperature. A blank sample was prepared with distilled water (2 mL). The absorbance of the solution was determined on a spectrophotometer at 765 nm. Measurements were performed in triplicates.

### 4.6. Determination of Monomeric Anthocyanin Content

The total monomeric anthocyanin contents were determined using the pH-differential method, which relies on the reversible structural transformation of the anthocyanin chromophore at different pH levels. This transition results in a distinct change in the absorbance spectrum, allowing for a rapid and precise quantification of anthocyanins even in the presence of interfering substances, such as polymerized or degraded pigments. Analyses were performed following the procedure described by Giusti and Wrolstad [42], in which 0.2 mL of the extract was mixed with 2.8 mL of two separate buffer systems: 0.025 M potassium chloride (pH 1.0) and 0.4 M sodium acetate (pH 4.5). After an incubation period of 15 min, the absorbance was measured at 515 nm and 700 nm using a spectrophotometer (Cary 60, Agilent Technologies, Santa Clara, CA, USA), with distilled water serving as the blank.

The anthocyanin content was calculated according to the following formula:

A = (A_λvis_ − A_700_)_pH1.0_ − (A_λvis_ − A_700_)_pH4.5_

The monomeric anthocyanin content (MA) was calculated according to the following formula:

MA (mg/kg) = (A × MW × DF × 1000) ÷ (ε × l)

where AC was expressed through cyanidin-3-glucoside equivalents, MW was the molecular weight of cyanidin-3-glucoside (449.2), DF was the dilution factor, ε was the molar absorptivity (26900) and l was the cuvette length (1 cm). All measurements were done in triplicate.

### 4.7. Determination of Proanthocyanidins

Briefly, 0.1 mL of the sample was pipetted and 1 mL of 4-dimethyl-aminocinnamaldehyde solution was added. The reaction mixture was left to stand for 30 min and the absorbance at 640 nm was measured. The result was recalculated from the calibration curve for procyanidin B2. All measurements were done in triplicate.

### 4.8. Adsorption Capacity

Adsorption capacity was calculated for total phenolics, proanthocyanidins, monomeric anthocyanins and all individual polyphenols. Total phenolics, proanthocyanidins and monomeric anthocyanins were determined spectrophotometrically in juice and supernatant, while individual polyphenols were determined by HPLC. Based on the difference between contents in juice and supernatant, the adsorption capacity for the parameters mentioned was expressed per gram of cellulose.

### 4.9. Determination of Individual Phenols Using High Performance Liquid Chromatography

Contents of individual phenolic compounds were determined using a 1260 Infinity II HPLC system (Agilent technology, Santa Clara, CA, USA). The mentioned system consisted of the following parts: a quaternary pump, a diode array detector and a Poroshell 120 EC-C 18 column (4.6 × 100 mm, 2.7 μm). The following were selected as mobile phases: 0.1% H_3_PO_4_ (A) and 100% methanol (B). The amount of injected sample was 20 μL, and the flow rate was set at 1 mL/min. Before injecting into the system, all samples were filtered using a PTFE filter (Macherey-Nagel, Düran, Germany) with a 0.45 µm pore size. The following conditions were used to elute the components: 0–38 min from 3% to 65% B and 38–45 min for 65% B. To prepare the calibration curves, concentrated standard solutions prepared in acidified methanol (1:99 HCl:methanol) were used for anthocyanins or in 100% methanol for other phenols. Calibration curves were made for the following components: chlorogenic acid (1–150 mg/L; r^2^ = 0.9993), neochlorogenic acid (1–150 mg/L; r^2^ = 0.9999), rutin (1–150 mg/L; r^2^ = 0.999), quercetin (1–150 mg/L; r^2^ = 0.9999), kaempferol (0.5–150 mg/L; r^2^ = 0.9997), and cyanidin-3-rutinoside (1–150 mg/L; r^2^ = 0.9997). Cyanidin-3-glucosyl-rutinoside was calculated using cyanidin-3-rutinoside. The UV/Vis spectrum was recorded in the range from 190 to 600 nm. Anthocyanins were identified at 520 nm, hydroxycinnamic acids at 320 nm, and flavonols at 360 nm. The samples were analyzed in parallel.

### 4.10. Determination of Antioxidant Activity

The antioxidant potential of the C/TC_Es was evaluated using four distinct spectrophotometric assays: DPPH, ABTS, FRAP, and CUPRAC. The DPPH radical scavenging capacity was determined based on the procedure by Brand-Williams et al. [43], with minor adjustments; 0.2 mL of the sample was combined with 3 mL of a 4 mM DPPH solution, and the absorbance was recorded at 517 nm after 15 min. For the ABTS radical cation assay, following the method of Arnao et al. [44], 0.2 mL of extract reacted with 3.2 mL of 7 mM ABTS reagent in the dark for 95 min, followed by absorbance measurement at 734 nm. The cupric-reducing antioxidant capacity (CUPRAC) was assessed according to Apak et al. [34]. The reaction mixture consisted of copper chloride, neocuproine, and ammonium acetate buffer (pH 7.0) in a 1:1:1 ratio. Specifically, 1 mL of each reagent was mixed with 0.2 mL of sample and 0.9 mL of distilled water, incubated for 30 min, and measured at 450 nm. Lastly, the ferric reducing antioxidant power (FRAP) was measured as described by Benzie and Strain [45]. A 0.2 mL aliquot of the sample was mixed with 3 mL of FRAP reagent and incubated for 30 min prior to reading the absorbance at 593 nm. In all assays, water was used as a blank, and analyses were performed in triplicate. The results are reported in micromoles of Trolox equivalent per 100 g of sample (µmol TE/100 g).

### 4.11. Determination of Color Parameters

Color properties and their subsequent variations were evaluated using a Minolta CR-400 chromameter (Konica Minolta, Tokyo, Japan). Results were expressed according to the CIELAB color space by recording lightness (L*), chromaticity coordinates a* (redness (+) and greenness (−)) and b* (yellowness (+) and blueness (−)), alongside chroma (C*) and hue angle (◦h). The total color difference (∆E) was calculated based on the obtained L*, a* and b* values. All measurements were performed in triplicate.

### 4.12. FTIR-ATR Spectroscopy

Fourier transform infrared spectroscopy with attenuated total reflection (FTIR-ATR) was employed to record the infrared (IR) spectra of pure cellulose and all prepared encapsulates. Measurements were conducted using a Cary 630 spectrometer (Agilent Technologies, Santa Clara, CA, USA) equipped with MicroLab Expert software. The analysis was performed over a spectral range from 4000 to 600 cm^−1^.

### 4.13. Statistical Data Processing

Statistical processing of the experimental data was performed using STATISTICA 13.1 (StatSoft Inc., Tulsa, OK, USA). The results were evaluated via analysis of variance (ANOVA), followed by Fisher’s least significant difference (LSD) test to determine significant differences at a level of *p* < 0.05. Data are expressed as mean values ± standard deviation. Additional processing of experimental data was conducted using OriginPro 2016 (OriginLab Corporation, Northampton, MA, USA) software for PCA analysis and heatmaps evaluation.

## 5. Conclusions

In conclusion, cellulose has been demonstrated as an effective delivery system for tart cherry phenolic compounds. The adsorption efficiency was strongly influenced by both cellulose content and complexation time, with optimal results achieved using 2.5% cellulose and a 15-min complexation period. This specific preparation exhibited the highest adsorption of total phenols, anthocyanins (notably cyanidin-3-rutinoside), and proanthocyanidins, while providing the strongest antioxidant activity. In stored encapsulates, C/TC_E_2.5% (15 min), the highest retention of total phenols and proanthocyanidins was observed, while C/TC_E_7.5% (60 min) best preserved anthocyanins. The developed cellulose-based encapsulates not only improved the stability of tart cherry phenolics but also helped preserve their antioxidant activity and maintained favorable color parameters, indicating minimal degradation of bioactive compounds. These results highlight the potential of cellulose-based encapsulates as natural additives for functional and innovative food formulations. Overall, these findings provide a solid basis for the application of plant-derived delivery systems in enhancing the quality, bioactivity, and shelf-life of phenolic-rich ingredients.

## Figures and Tables

**Table 1:** Phenolic compounds and antioxidant activity of tart cherry juice.

**Spectrophotometric Analysis**	
Total phenolics (g/L)	6.58 ± 0.25
Proanthocyanidins (mg/L)	1255.12 ± 1.38
Monomeric anthocyanins (mg/L)	469.39 ± 0.99
**HPLC Analysis**	
Cyanidin-3-glucosyl-rutinoside (µg/mL)	344.29 ± 0.78
Cyanidin-3-rutinoside (µg/mL)	423.71 ± 1.05
Neochlorogenic acid (µg/mL)	105.92 ± 0.89
Chlorogenic acid (µg/mL)	173.42 ± 0.75
Rutin (µg mL)	78.54 ± 0.39
Quercetin (µg/mL)	46.25 ± 0.28
Kaempferol (µg/mL)	31.23 ± 0.41
**Antioxidant Activity**	
DPPH (µmol TE/100 mL)	36.51 ± 0.78
ABTS (µmol TE/100 mL)	27.65 ± 0.87
FRAP (µmol TE/100 mL)	5.72 ± 0.82
CUPRAC (µmol TE/100 mL)	199.56 ± 1.36

**Table 2:** Total phenolic content (g/kg) in cellulose/tart cherry encapsulates (C/TC_Es) complexed for 15 or 60 min, after preparation and after 12 months of storage.

Samples	Complexation Time (Minutes)	
	15	60
	After preparation	
C/TC_E_2.5%	4.26 ± 0.15 ^a^	3.94 ± 0.33 ^a^
C/TC_E_5%	4.04 ± 0.04 ^a,b^	3.79 ± 0.34 ^a^
C/TC_E_7.5%	3.93 ± 0.10 ^b^	3.46 ± 0.12 ^a,b^
C/TC_E_10%	3.14 ± 0.05 ^c^	3.26 ± 0.13 ^b^
After 12 months of storage		
C/TC_E_2.5%	3.99 ± 0.05 ^b^	3.22 ± 0.07 ^b^
C/TC_E_5%	3.11 ± 0.12 ^c^	3.28 ± 0.06 ^b^
C/TC_E_7.5%	3.18 ± 0.13 ^c^	2.85 ± 0.02 ^c^
C/TC_E_10%	2.92 ± 0.03 ^c^	2.68 ± 0.05 ^d^

Statistical variations are marked with different letters (a–d) within each column, signifying differences at *p* ≤ 0.05. The percentages 2.5–10% represent the cellulose content in C/TC_Es samples.

**Table 3:** Total anthocyanins content (mg/kg) in cellulose/tart cherry encapsulates (C/TC_Es) complexed for 15 or 60 min, after preparation and after 12 months of storage.

Samples	Complexation Time (Minutes)	
	15	60
	After preparation	
C/TC_E_2.5%	354.25 ± 1.21 ^a^	301.12 ± 2.74 ^a^
C/TC_E_5%	321.21 ± 2.41 ^b^	281.32 ± 1.87 ^b^
C/TC_E_7.5%	308.21 ± 2.47 ^c^	242.32 ± 1.54 ^c^
C/TC_E_10%	294.25 ± 1.42 ^d^	228.21 ± 1.11 ^d^
After 12 months of storage		
C/TC_E_2.5%	212.49 ± 1.21 ^e^	189.70 ± 1.54 ^e^
C/TC_E_5%	181.07 ± 1.36 ^f^	169.43 ± 1.45 ^f^
C/TC_E_7.5%	175.05 ± 1.50 ^g^	158.18 ± 1.36 ^g^
C/TC_E_10%	164.98 ± 0.98 ^h^	127.29 ± 2.21 ^h^

Statistical variations are marked with different letters (a–h) within each column, signifying differences at *p* ≤ 0.05. The percentages 2.5–10% represent the cellulose content in C/TC_Es samples.

**Table 4:** Total proanthocyanidins content (mg/kg) in cellulose/tart cherry encapsulates (C/TC_Es) complexed for 15 or 60 min, after preparation and after 12 months of storage.

Samples	Complexation Time (Minutes)	
	15	60
	After preparation	
C/TC_E_2.5%	681.84 ± 1.56 ^a^	642.21 ± 7.05 ^a^
C/TC_E_5%	567.51 ± 4.63 ^b^	528.52 ± 2.92 ^b^
C/TC_E_7.5%	448.91 ± 2.46 ^d^	414.67 ± 2.96 ^d^
C/TC_E_10%	358.45 ± 0.51 ^e^	306.29 ± 3.31 ^f^
After 12 months of storage		
C/TC_E_2.5%	478.34 ± 1.75 ^c^	434.39 ± 2.44 ^c^
C/TC_E_5%	359.67 ± 1.82 ^e^	331.19 ± 1.29 ^e^
C/TC_E_7.5%	268.59 ± 2.25 ^f^	228.94 ± 1.28 ^g^
C/TC_E_10%	201.23 ± 0.45 ^g^	190.43 ± 1.14 ^h^

Statistical variations are marked with different letters (a–h) within each column, signifying differences at *p* ≤ 0.05. The percentages 2.5–10% represent the cellulose content in C/TC_Es samples.

**Table 5:** Concentrations of individual phenolic compounds (µg/g) in cellulose/tart cherry encapsulates (C/TC_Es) complexed for 15 min, measured after preparation and after 12 months of storage.

Phenol Compound	C/TC_E_2.5%	C/TC_E_5%	C/TC_E_7.5%	C/TC_E_10%
	15 min of Complexation			
	After Preparation			
Cyanidin-3-glucosyl-rutinoside	76.01 ± 1.03 ^a^	65.94 ± 3.17 ^b^	65.00 ± 0.97 ^b^	57.37 ± 1.23 ^c^
Cyanidin-3-rutinoside	185.89 ± 1.78 ^a^	171.73 ± 3.71 ^b^	156.82 ± 0.68 ^c^	147.06 ± 1.41 ^d^
Neochlorogenic acid	87.03 ± 0.59 ^a^	84.54 ± 1.56 ^b^	76.70 ± 0.56 ^c^	74.09 ± 0.54 ^d^
Chlorogenic acid	151.71 ± 0.64 ^a^	141.99 ± 2.55 ^b^	126.65 ± 2.89 ^c^	127.00 ± 0.17 ^c^
Rutin	54.49 ± 0.68 ^a^	52.17 ± 1.18 ^a^	48.45 ± 0.27 ^b^	46.01 ± 0.24 ^c^
Quercetin	19.92 ± 0.26 ^a^	19.43 ± 0.53 ^a^	18.47 ± 0.11 ^b^	18.56 ± 0.19 ^b^
Kaempferol	9.40 ± 0.12 ^a^	8.94 ± 0.15 ^b^	8.50 ± 0.07 ^c^	8.47 ± 0.01 ^c^
	After 12 months of storage			
Cyanidin-3-glucosyl-rutinoside	32.31 ± 0.06 ^a^	27.14 ± 0.97 ^b^	24.32 ± 0.09 ^c^	21.28 ± 0.70 ^d^
Cyanidin-3-rutinoside	87.83 ± 0.13 ^a^	70.39 ± 0.36 ^b^	64.34 ± 1.03 ^c^	53.88 ± 0.11 ^d^
Neochlorogenic acid	76.00 ± 0.48 ^a^	72.50 ± 0.74 ^b^	65.11 ± 1.87 ^c^	58.73 ± 0.43 ^d^
Chlorogenic acid	136.14 ± 1.19 ^a^	133.46 ± 1.49 ^a^	116.76 ± 0.67 ^b^	103.45 ± 2.77 ^c^
Rutin	49.18 ± 0.81 ^a^	45.41 ± 0.07 ^b^	41.95 ± 0.69 ^c^	39.30 ± 0.03 ^d^
Quercetin	22.19 ± 0.12 ^a^	21.60 ± 0.14 ^b^	19.99 ± 0.29 ^c^	20.01 ± 0.11 ^c^
Kaempferol	9.69 ± 0.04 ^a^	9.22 ± 0.02 ^b^	8.56 ± 0.15 ^c^	8.36 ± 0.02 ^c^

Statistical variations are marked with different letters (a–d) within each row, signifying differences at *p* ≤ 0.05. The percentages 2.5–10% represent the cellulose content in C/TC_Es samples.

**Table 6:** Concentrations of individual phenolic compounds (µg/g) in cellulose/tart cherry encapsulates (C/TC_Es) complexed for 60 min, measured after preparation and after 12 months of storage.

Phenol Compound	C/TC_E_2.5%	C/TC_E_5%	C/TC_E_7.5%	C/TC_E_10%
	60 min of Complexation			
	After Preparation			
Cyanidin-3-glucosyl-rutinoside	53.80 ± 1.12 ^a^	49.95 ± 0.39 ^b^	44.11 ± 0.48 ^c^	50.77 ± 0.86 ^b^
Cyanidin-3-rutinoside	135.66 ± 4.34 ^a^	133.58 ± 1.86 ^a^	110.48 ± 0.66 ^c^	117.26 ± 0.46 ^b^
Neochlorogenic acid	73.64 ± 1.73 ^a^	73.27 ± 0.32 ^a^	64.95 ± 0.73 ^b^	64.06 ± 0.18 ^b^
Chlorogenic acid	127.21 ± 2.02 ^a^	125.21 ± 1.87 ^a^	113.68 ± 0.57 ^b^	111.99 ± 1.59 ^b^
Rutin	43.63 ± 0.52 ^a^	42.56 ± 0.26 ^a^	40.08 ± 0.00 ^b^	38.48 ± 0.37 ^c^
Quercetin	23.21 ± 0.45 ^a^	23.16 ± 0.13 ^a^	20.98 ± 0.06 ^b^	21.34 ± 0.09 ^b^
Kaempferol	9.79 ± 0.26 ^a^	9.75 ± 0.03 ^a^	8.96 ± 0.07 ^b^	8.98 ± 0.03 ^b^
	After 12 months of storage			
Cyanidin-3-glucosyl-rutinoside	23.02 ± 0.63 ^a^	21.97 ± 1.36 ^a,b^	21.45 ± 0.26 ^b^	20.79 ± 0.06 ^c^
Cyanidin-3-rutinoside	55.02 ± 0.32 ^a^	49.67 ± 1.13 ^b^	49.00 ± 0.10 ^b^	46.32 ± 1.99 ^c^
Neochlorogenic acid	62.44 ± 0.33 ^a^	58.18 ± 1.38 ^b^	56.37 ± 0.44 ^b^	53.45 ± 1.43 ^c^
Chlorogenic acid	106.93 ± 2.23 ^b^	113.43 ± 0.86 ^a^	104.63 ± 2.51 ^b^	96.39 ± 0.45 ^c^
Rutin	40.90 ± 0.27 ^a^	37.85 ± 0.48 ^b^	37.24 ± 0.14 ^b^	35.88 ± 0.64 ^c^
Quercetin	24.49 ± 0.08 ^a^	24.49 ± 0.14 ^a^	23.18 ± 0.13 ^b^	22.34 ± 0.01 ^c^
Kaempferol	9.40 ± 0.02 ^a^	9.51 ± 0.12 ^a^	9.11 ± 0.07 ^b^	8.86 ± 0.17 ^c^

Statistical variations are marked with different letters (a–c) within each row, signifying differences at *p* ≤ 0.05. The percentages 2.5–10% represent the cellulose content in C/TC_Es samples.

**Table 7:** Antioxidant activity (µmol TE/100 g) of cellulose/tart cherry encapsulates (C/TC_Es) complexed for 15 or 60 min after preparation and after 12 months of storage.

	DPPH		ABTS		FRAP		CUPRAC	
Samples	Complexation Time (Minutes)							
	15	60	15	60	15	60	15	60
After preparation								
C/TC_E_2.5%	26.98 ± 0.22 ^a^	30.24 ± 0.18 ^a^	18.98 ± 0.29 ^a^	17.88 ± 0.12 ^a^	3.04 ± 0.05 ^a^	2.70 ± 0.11 ^a^	167.58 ± 2.01 ^a^	151.63 ± 1.24 ^a^
C/TC_E_5%	25.51 ± 0.29 ^b^	28.85 ± 0.30 ^b^	16.78 ± 0.31 ^b^	17.63 ± 0.31 ^a^	2.75 ± 0.13 ^b^	2.69 ± 0.09 ^a^	151.90 ± 2.35 ^b^	142.35 ± 4.35 ^b^
C/TC_E_7.5%	25.16 ± 0.36 ^b,c^	28.63 ± 0.94 ^b^	15.74 ± 0.30 ^c^	14.95 ± 0.20 ^b^	2.69 ± 0.11 ^b^	2.28 ± 0.06 ^b^	138.97 ± 1.57 ^c^	131.26 ± 4.51 ^c^
C/TC_E_10%	25.72 ± 0.33 ^b^	27.92 ± 0.36 ^b^	13.85 ± 0.13 ^d^	14.77 ± 0.16 ^b^	2.33 ± 0.15 ^c^	2.23 ± 0.11 ^b^	125.69 ± 0.23 ^d^	128.70 ± 2.20 ^c^
After storage								
C/TC_E_2.5%	24.78 ± 0.48 ^c,d^	24.77 ± 0.59 ^c^	15.41 ± 0.24 ^c^	12.58 ± 0.35 ^c^	2.31 ± 0.07 ^c^	2.27 ± 0.06 ^b^	138.41 ± 0.12 ^c^	128.85 ± 0.05 ^c^
C/TC_E_5%	24.64 ± 0.17 ^c,d^	24.99 ± 0.47 ^c^	13.21 ± 0.23 ^d^	12.09 ± 0.32 ^c^	1.98 ± 0.17 ^d^	1.94 ± 0.23 ^c^	122.26 ± 0.31 ^e^	126.31 ± 5.04 ^c^
C/TC_E_7.5%	24.12 ± 0.30 ^d^	24.49 ± 0.23 ^c^	12.12 ± 0.08 ^e^	11.04 ± 0.19 ^d^	1.67 ± 0.06 ^d^	1.68 ± 0.04 ^c^	113.30 ± 1.97 ^f^	107.49 ± 1.43 ^d^
C/TC_E_10%	23.48 ± 0.77 ^e^	24.21 ± 0.97 ^c^	11.43 ± 0.36 ^f^	10.30 ± 0.63 ^d^	1.63 ± 0.10 ^d^	1.56 ± 0.18 ^c^	104.73 ± 2.44 ^g^	101.16 ± 0.08 ^d^

Statistical variations are marked with different letters (a–g) within each column, signifying differences at *p* ≤ 0.05. The percentages 2.5–10% represent the cellulose content in C/TC_Es samples.

**Table 8:** Color parameters of cellulose/tart cherry encapsulates (C/TC_Es) complexed for 15 and 60 min, after preparation.

Cellulose Content	L*	a*	b*	ΔE	°h	C*
100%	93.40 ± 0.00	0.42 ± 0.02	6.65 ± 0.01		86.34 ± 0.11	6.66 ± 0.01
	15 min of complexation					
C/TC_E_2.5%	66.98 ± 0.03 ^f^	29.10 ± 0.07 ^a^	2.36 ± 0.06 ^c^	39.23	4.63 ± 0.12 ^e^	29.16 ± 0.07 ^a^
C/TC_E_5%	68.16 ± 0.01 ^e^	28.59 ± 0.03 ^b^	2.04 ± 0.02 ^e^	38.11	4.08 ± 0.04 ^f^	28.66 ± 0.03 ^b^
C/TC_E_7.5%	68.40 ± 0.02 ^d^	28.30 ± 0.05 ^c^	1.63 ± 0.04 ^f^	37.78	3.31 ± 0.08 ^g^	28.34 ± 0.04 ^c^
C/TC_E_10%	70.64 ± 0.02 ^b^	27.00 ± 0.03 ^e^	1.39 ± 0.03 ^g^	35.39	2.94 ± 0.07 ^g^	27.04 ± 0.03 ^e^
	60 min of complexation					
C/TC_E_2.5%	68.14 ± 0.01 ^e^	27.38 ± 0.03 ^d^	3.24 ± 0.02 ^a^	37.10	6.75 ± 0.05 ^b^	27.57 ± 0.03 ^d^
C/TC_E_5%	68.89 ± 0.01 ^d^	26.53 ± 0.03 ^f^	3.29 ± 0.01 ^a^	35.96	7.08 ± 0.04 ^a^	26.73 ± 0.03 ^f^
C/TC_E_7.5%	69.39 ± 0.01 ^c^	26.23 ± 0.02 ^g^	2.92 ± 0.02 ^b^	35.45	6.35 ± 0.05 ^c^	26.39 ± 0.02 ^g^
C/TC_E_10%	71.85 ± 0.01 ^a^	25.30 ± 0.00 ^h^	2.13 ± 0.02 ^d^	33.23	4.81 ± 0.03 ^d^	25.39 ± 0.01 ^h^

Statistical variations are marked with different letters (a–h) within each column, signifying differences at *p* ≤ 0.05. The percentages 2.5–10% represent the cellulose content in C/TC_Es samples.

**Table 9:** Color parameters of cellulose/tart cherry encapsulates C/TC_Es complexed for 15 or 60 min, after storage.

Cellulose Content	L*	a*	b*	ΔE	°h	C*
100%	93.40 ± 0.0	0.42 ± 0.02	6.65 ± 0.01		86.34 ± 0.11	6.66 ± 0.01
	15 min of complexation					
C/TC_E_2.5%	69.75 ± 0.02 ^h^	19.21 ± 0.02 ^a^	9.67 ± 0.01 ^c^	12.61	26.72 ± 0.03 ^e^	21.50 ± 0.02 ^a^
C/TC_E_5%	71.80 ± 0.05 ^f^	17.83 ± 0.04 ^b^	9.42 ± 0.01 ^d^	13.55	27.85 ± 0.08 ^d^	20.17 ± 0.03 ^c^
C/TC_E_7.5%	73.16 ± 0.01 ^d^	17.76 ± 0.04 ^b^	8.42 ± 0.03 ^f^	13.41	25.37 ± 0.13 ^f^	19.66 ± 0.03 ^d^
C/TC_E_10%	73.58 ± 0.02 ^b^	16.77 ± 0.03 ^d^	8.99 ± 0.03 ^e^	13.08	28.18 ± 0.11 ^d^	19.02 ± 0.02 ^f^
	60 min of complexation					
C/TC_E_2.5%	70.96 ± 0.01 ^g^	17.28 ± 0.03 ^c^	10.77 ± 0.01 ^a^	12.91	31.94 ± 0.07 ^c^	20.36 ± 0.02 ^b^
C/TC_E_5%	72.13 ± 0.01 ^e^	16.40 ± 0.03 ^e^	10.61 ± 0.02 ^a^	12.91	32.91 ± 0.10 ^a^	19.54 ± 0.02 ^e^
C/TC_E_7.5%	73.46 ± 0.01 ^c^	15.96 ± 0.02 ^f^	10.20 ± 0.01 ^b^	13.23	32.59 ± 0.06 ^b^	18.94 ± 0.01 ^f^
C/TC_E_10%	74.39 ± 0.00 ^a^	15.53 ± 0.02 ^g^	9.72 ± 0.01 ^c^	12.63	32.04 ± 0.04 ^c^	18.33 ± 0.01 ^g^

Statistical variations are marked with different letters (a–h) within each column, signifying differences at *p* ≤ 0.05. The percentages 2.5–10% represent the cellulose content in C/TC_Es samples.

## Data Availability

The original contributions presented in this study are included in the article. Further inquiries can be directed to the corresponding author.
